# Clipping Effect on the Grain Nitrogen and Protein Fractions of Ancient and Old Wheats Grown in a Mediterranean Environment

**DOI:** 10.3390/foods12132582

**Published:** 2023-07-02

**Authors:** Marina Mefleh, Rosella Motzo, Fatma Boukid, Francesco Giunta

**Affiliations:** 1 Department of Agricultural Sciences, University of Sassari, Viale Italia 39/a, 07100 Sassari, Italy; motzo@uniss.it (R.M.); giunta@uniss.it (F.G.); 2 ClonBio Group Ltd., 6 Fitzwilliam Pl, D02 XE61 Dublin, Ireland; fboukid@clonbioeng.com

**Keywords:** clipping, grain nitrogen, gluten, protein composition

## Abstract

This study is the first to assess the effects of clipping, cultivar, season, and their interactions on the protein composition of six old and ancient wheat cultivars (n = 6). For this, nitrogen content, the proportion of wheat protein fractions, and the molecular weight distribution of the extractable and unextractable glutenin polymers were investigated as a function of cultivar and clipping in two consecutive seasons. The relationships between genotypic variation in grain nitrogen and protein fraction content under clipping and non-clipping conditions were also assessed. Clipping delayed and shortened the grain filling period of all of the cultivars. The protein composition of some cultivars behaved differently to clipping due to differences in the environmental conditions of S1 (exceptional dry season) and S2 (rainy season). In S1, clipping decreased the ratio of gliadins over glutenins (GLI/GLU) (<1) of Cappelli and Giovanni Paolo, while in S2, clipping improved the GLI/GLU of Giovanni Paolo, Monlis, and Norberto. The unextractable polymeric proteins were not affected by clipping. Khorasan was shown to be indifferent to clipping in S1 and S2. These results suggest that it is possible to have ancient/old wheats suitable for a dual-purpose system, in different climatic conditions, while maintaining good grain quality traits. The increased market demand for ancient and old wheats presents an economic opportunity for farmers who adopt the dual-purpose technique to cultivate these resilient crops again and increase their profit margins and revenues.

## 1. Introduction

Ancient wheat species (einkorn, emmer, and spelt) were grown by ancient civilization and represent a transition from wild wheats (before domestication) to the landraces and old cultivars of durum and bread wheat. Ancient wheats are characterized by hulled and small kernels. On the other hand, landraces and old cultivars of durum wheat are free-threshed, generally tall of the cultivars, cultivated before the introgression of the dwarfing *Rht-1* genes. The cultivation of ancient and old wheats decreased gradually after the Green Revolution to be replaced by the high-yielding modern semi-dwarf wheat cultivars [1]. Ancient and old wheats were therefore limited to marginal low-fertility areas where the performance of the high-yielding modern cultivars could not be achieved [2,3]. In fact, under low-input conditions, the yield of some old durum wheat cultivars has been shown to not be different from that of modern durum wheat cultivars, with old cultivars having even higher grain nitrogen content [4]. In general, the intensive cultivation of modern wheat cultivars resulted in a quantum increase in wheat grain production worldwide. Nevertheless, adopting modern cultivars resulted in wheat biodiversity loss. During the last two decades, there was increased public awareness toward the introduction of sustainable development policy and programs such as the 2030 Agenda [5]. The Sustainable Development Goals 2 and 15 are indeed devoted to the promotion of sustainable agriculture and the protection and restoration of biodiversity loss [5]. In this frame, reintegrating the cereal biodiversity of landraces and old varieties into the low-input cropping systems would play a crucial role as a source of allelic variation related to yield, grain quality, and low-input adaptability [6,7]. Moreover, reducing the input costs contributes to improved profit margins and the overall economic viability of agricultural operations, while adopting a dual-purpose crops system could be a strategy in promoting sustainability and resilience of the farming system.

Ancient and old wheat plants are tall in height and produce high vegetative biomass [8,9]. These vegetative biomasses can be grazed by sheep and cattle during their growth in many farming systems [10]. This would contribute to assuring a continuous seasonal supply of herbage to animals during critical periods (e.g., periods of low forage availability) [11] without renouncing the grains in the same season (dual-purpose utilization). Grazing the herbage before the onset of stem elongation can exert several positive effects on old and ancient wheat crops, such as reducing both the incidence of lodging [12,13] and the water used early in the season. As such, this practice increases water use efficiency during anthesis and the grain filling period (GFP) [14,15]. Tall of the cultivars are particularly suited to this type of utilization because they suffer less from grain yield reduction following grazing compared to high-yielding improved semi-dwarf cultivars [16].

In particular, Italian ancient and old durum wheats were found to be suitable for dual-purpose utilization [8,13]. Thus, adopting mixed farming systems of ancient and old grains can increase the economic advantages of farmers in the Mediterranean environment. The dual-purpose utilization of Italian old and ancient wheats was reported to make herbage available to animals in the critical winter period, without decreasing the grain yield attainable after grazing in the same season [8,13]. However, it is still uncertain as to whether clipping affects the wheat grain protein composition or not. These latter traits are the main ones behind the wheat end-use quality and type (e.g., pasta, bakery, or pastry).

Extractable grain nitrogen (EGN) is the content of nitrogen allocated to the metabolic (albumin and globulins) and the SDS-extractable storage proteins (gliadins and glutenins) during the development and the maturation of the grain, while unextractable grain nitrogen is allocated to the SDS-unextractable polymeric proteins. These latter ones are formed via the aggregation of the glutenins, mainly the low-molecular-weight glutenin subunits (LMW-GSs), which by having a greater amount of free SH groups undergo redox change simultaneously with continuous grain dehydration [17]. The proportion of the different wheat protein classes, the molecular weight distribution of the glutenin polymers, and the percentage of the unextractable polymeric protein (UPP%) are relevant indicators to predict the rheological properties of dough [4,18] and the quality of wheat-based products [19].

In a previous study of our group, the clipping of a set of ancient species and old wheat cultivars delayed the flowering time to different extents depending on the cultivar and reduced the total nitrogen (N) present in the crop at anthesis and the grain protein percentage and content [10]. The present study aims to go further in investigating the effect of clipping on the protein composition of the same set of wheat. To the best of our knowledge, this study is the first to investigate the effect of clipping, cultivar, season, and their interactions on the protein composition of six wheat cultivars belonging to four species of the genus Triticum (emmer, einkorn, durum, and turanicum).

## 2. Material and Methods

### 2.1. Site, Soil, and Agronomic Management

A two-year trial was conducted during the 2017/2018 and 2018/2019 periods at the Ottava experimental station (41° N; 8° E; 80 m a.s.l) of the University of Sassari. The station is situated in a Mediterranean environment with an average annual rainfall of 539 mm, primarily occurring between October and April. The soil contains a layer of limestone at a depth of 0.4 to 0.5 m, has 45 kg ha^−1^ of mineral nitrogen, 1.4 ± 0.3% soil organic matter content, 40 ± 4.4% total CaCO3 content, and 8.4 ± 0.5 ppm available phosphorus. The fertilization process involved two separate applications. At sowing, nitrogen and phosphorous were applied in the form of diammonium phosphate at rates of 36 kg ha^−1^ of N and 92 kg ha^−1^ of P2O5. Following clipping, 26 kg ha^−1^ of ammonium nitrate was applied (2nd application) to both the clipped and non-clipped plots. The previous crop was faba bean in both seasons, and a seedbed was prepared by ploughing the soil to a depth of 0.25 m followed by surface cultivation. Weeds, pests, and diseases were controlled using chemical treatments. The sowing rate was set to 200 viable seeds per m^2^. The experimental fields were sown on 25 and 26 October in the first and second season, respectively.

The field experiments analyzed in this study were also the subject of the work published by [8] on grain yield and the suitability of ancient wheat species to dual-purpose utilization.

### 2.2. Factors, Treatment, and Design of the Experiment

The field experiment was laid out in four blocks as a split-plot. The cultivar factor was assigned to the main plot, while the clipping factor was assigned to the subplot. Each plot measured 10 m^2^ and contained 6 rows that were 8.4 m long, with spacing of 0.15 m between rows. Two fields were used in the study over the two-year period. Clipping was performed using a lawn mower when the terminal spikelet stage was detected, limiting the aboveground plant height to a maximum of 2 cm in the ‘clipped’ treatment. The six wheat cultivars used in the study (Table 1) were ‘Monlis’ and ‘Norberto’, two einkorn cultivars, ‘Padre Pio’ and ‘Giovanni Paolo’, two improved emmer cultivars selected for their adaptability and resistance to diseases and lodging, an old durum wheat cultivar called ‘Senatore Cappelli’, and an old turanicum wheat variety called ‘Khorasan’. The experiment was conducted in the two seasons of 2016/17 (S1) and 2017/18 (S2).

All of the agronomic measurements performed in this study (emergence, flag leaf appearance, anthesis, physiological maturity, number of leaves, lodging incidence, and biomass samplings at terminal spikelet, anthesis, and maturity) were described in [8]. As already mentioned in [8], one of the limitations of the use of old and ancient cultivars is their high susceptibility to lodging. Clipping reduced but did not eliminate lodging, which caused some technical problems at harvesting.

### 2.3. Meteorological Data

Meteorological data such as maximum and minimum temperatures, rainfall, solar radiation, and air relative humidity were monitored by a meteorological station approximately 300 m away from the field. Data were used to calculate the minimum and maximum temperatures and the number of days with temperatures higher than 25 °C and 30 °C.

### 2.4. Grain Weight and Nitrogen Content

Grain moisture content and grain weight were obtained from four 250-grain subsamples per plot. Nitrogen percentage was determined on each subsample by means of a Carbon/Hydrogen/Nitrogen Analyzer (628 Series, LECO Corporation, St. Joseph, MI, USA) [19]. The combustion temperature was set at 1050 °C. Nitrogen data were used to calculate the amount of nitrogen (in µg) per grain (total grain N) as grain weight at dry basis × N percentage.

### 2.5. Protein Characterization

#### 2.5.1. Sequential Extraction of Gliadin and Glutenin for Reverse-Phase High-Performance Liquid Chromatography (RP-HPLC) Analysis

Albumin and globulin, gliadin, and glutenin extraction and quantification were performed as previously described [4]. The relative percentage of each protein fraction (albumin–globulin, gliadin, and glutenin) was calculated from the total protein, and the relative percentage of each glutenin subunit (HMW and LMW) was calculated from total gluten. The ratio of gliadin/glutenin was calculated as the ratio of the gliadin peak over the glutenin peak. The chromatograms of gliadins and glutenins are present in Figures S1 and S2 of the Supplementary Materials.

#### 2.5.2. Extraction of Extractable and Un-Extractable Polymeric Proteins for Size-Exclusion High-Performance Liquid Chromatography (SE-HPLC) Analysis

Protein extraction and quantification were performed as previously described by [4]. The total area under each chromatogram obtained from SDS-extractable and un-extractable protein extracts was expressed as a percentage (EP% and UP%, respectively) of the sum of the total area of both chromatograms. The un-extractable polymeric protein fraction (UPP%) was calculated as a percentage of the total polymeric protein (UPP% = UP%/(sum of glutenins% of EP) + UP%).

Grain nitrogen data and the percentages of protein fractions (determined using RP-HPLC) and the percentage of EP and UP (determined using SE-HPLC) were used to estimate the µg of nitrogen of each protein fraction and subunits and the nitrogen content of EP and UP [4]. From here on, the ug of nitrogen content of a fraction or subunit will simply be referred to as ‘content’; for example, the µg of nitrogen of gliadin will be referred to as ‘gliadin content’, as in Mefleh et al. [4].

### 2.6. Statistical Analysis

For each season × cultivar × clipping combination, grain samples of 200 g each from the four field blocks were grinded together for subsequent chemical analysis, which was conducted in triplicate. Data were analyzed using R software (R Core Team, 2014, Vienna, Austria). Factorial analysis of variance was conducted to evaluate the effect of clipping, cultivar, and season, as well as their interactions. The Pearson correlation coefficient was used to evaluate the existence of causal relationships between pairs of traits.

## 3. Results and Discussion

### 3.1. Weather Conditions and Phenology

**Figure 1:**
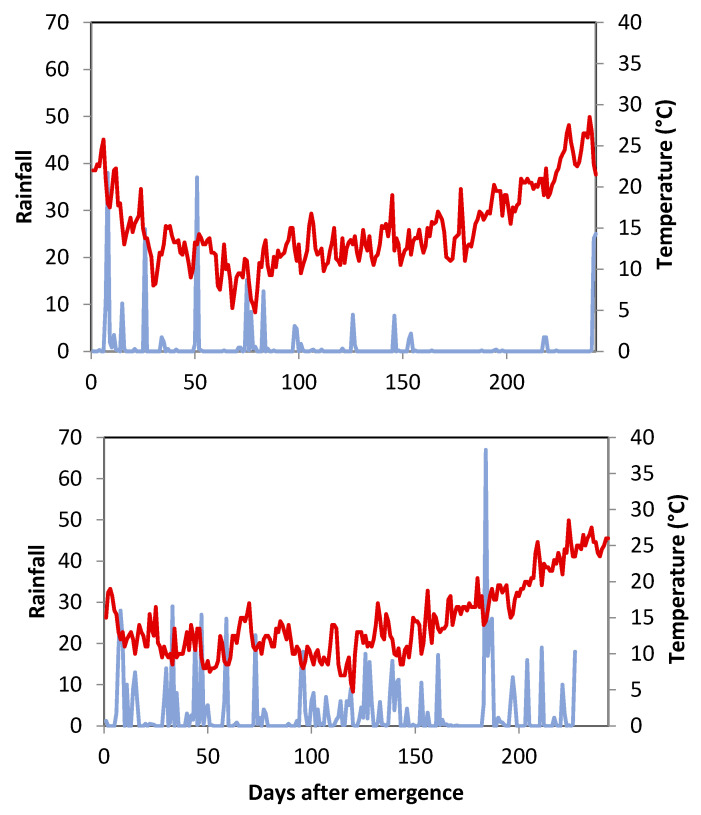
Weather for S1 (2016) (**upper panel**) and S2 (2017) (**lower panel**) from emergence (1st November) to maturity. Rainfall (blue solid lines) and mean air temperature (red line).

Weather conditions and phenology have been partly discussed in [8] with reference to their effects on biomass and grain yield under dual-purpose utilization. In this analysis, the focus is on the grain filling period (GFP), and hence on the weather conditions during the period when the nitrogen allocation to protein fractions is determined. The climatic conditions determined by the two seasons were markedly different in terms of pluviometry with regard to amount, distribution, and thermal regime, and thus affected water status and the response to temperature of the crop during both vegetative and reproductive phases differently (Figure 1). During S1, only 311 mm of rain fell between October and June, which represents 40% of the rainfall in S2 (785 mm). The GFPs of all of the cultivars were longer in S2 than in S1 due to the higher rainfall during S2. However, the GFP in S2 intercepted more days (34 days), with average temperatures above 25 °C, compared to S1 (24 days) (Figure 1).

**Figure 2:**
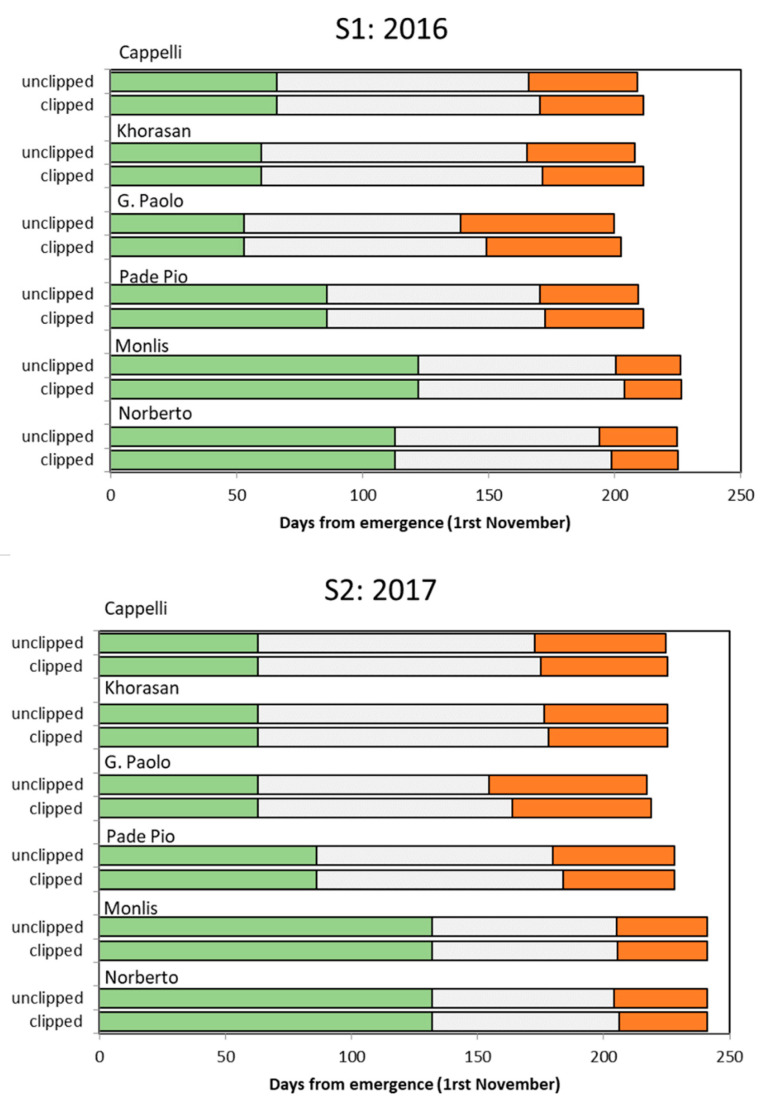
Effect of season, cultivar, and clipping on the length of the vegetative growth (from emergence until clipping, in green and from clipping until flowering, in white) and on the grain filling period (from flowering until physiological maturity, in orange).

The great phenological differences between cultivars were reflected in the different duration of the vegetative growth (that lasted until clipping) and in the start and end of the GFP (Figure 2). According to the duration of the grain filling period, roughly considered to be coincident with flowering–ripening, we can distinguish three different groups of cultivars: (a) Giovanni Paolo was the earliest in flowering and had the longest GFP (around 2 months), (b) Cappelli, Khorasan, and Padre Pio had a GFP of around one month and a half, and (c) Monlis and Norberto were characterized by being the latest in flowering and having the shortest GFP (around a month).

Clipping delayed flowering by 6 days on average in S1 and by 3 days in S2, with the greater delay (9–10 days) observed in Giovanni Paolo (Figure 2). On average, clipping shortened the GFP by 3.3 days in S1 and by 3.0 days in S2. However, the GFPs of all of the cultivars, except for Giovanni Paolo, were longer in S2 compared to S1 due the high rainfall of S2.

Cappelli, Khorasan, and Padre Pio suffered a rainfall deficit in S1 but not in S2, while the other cultivars suffered a rainfall deficit in both seasons (Table 2). In fact, Hinson et al. [20] found that limited early season precipitation resulted in slow forage growth in all years and inadequate forage production. The average of the minimum temperatures of S2 were higher than the ones of S1 for all of the cultivars. The late flowering of Monlis and Norberto combined with the delay induced by clipping resulted in grain filling taking place in the month of June, when crops were exposed to more days with temperatures between 25 and 30 °C (22 days compared to 10 and 15 for the other cultivars) and also to more days with maximum temperatures exceeding 30 °C (11 days compared to 4 and 2 for the other cultivars). In S2, the number of days with maximum temperatures exceeding 30 °C was lower than in S1.

### 3.2. Grain Nitrogen Content, Protein Composition, and Quality

EGNµg was significantly influenced by clipping, cultivar, and season, while their interactions did not show any significant effects (Table 3). Cultivar was the main factor impacting EGNµg. Giovanni Paolo and Khorasan had the highest EGNµg, almost double the values of Monlis and Norberto (Table 4). This can be related to differences in grain weight, i.e., 50 and 55 mg, for Giovanni Paolo and Khorasan, respectively, against 23 mg for Monlis and 26 mg for Norberto [8]. EGNµg was found to be higher during S2 compared to S1, and this might be due to favorable weather conditions for grain filling [21].

Table 3 shows that cultivar had a pronounced effect on UPP% and UP% compared to season. Clipping did not show a significant effect on UPP%, and UP%. Thus, it is expected that this treatment would have no effect on the formation of polymeric protein aggregates. The interactions did not show any significant effect on EGNµg, UPP%, and UP%.

Table 4 showed that a reduction in EGNµg was observed after clipping. This result is not consistent with studies conducted on the grain nitrogen of triticale [22], common wheat [23], and hard red and white winter wheats [24]. This discrepancy might be due to the use of different cultivars grown under different environmental conditions. In our study, EGNµg was not affected by the interaction of clipping × season. This contrasts the work of [23], whereby the authors reported contrasting effects of clipping on grain N content depending on the environmental conditions, with dry seasons resulting in greater N recovery after clipping, compared to favorable rainy seasons.

Regarding the cultivar effect (Table 4), Giovanni Paolo, Monlis, and Norberto evidenced the highest UP% and Giovanni Paolo had the highest UPP%, while Padre Pio had the lowest values. UPP% is strictly related to gluten strength and dough strength and stability [4]. All of the cultivars, except Padre Pio, had an UPP% higher than 20%, the threshold for weak dough [25], and can be classified within the category of ‘high UPP%’ according to [26]. On average, the UPP% of the set of wheats studied was higher than a previous study focusing on a set of 16 Italian durum wheat cultivars [4]. This can be related to species as well as varietal and/or environmental differences. Edwards et al. [27] showed that UPP% is directly associated with genes located on Glu-B1 known to encode HMW-GS. In particular, the weak versions (e.g., HMW-20) have the lowest UPP% compared with the strong versions (e.g., HMW-7+8, 6+8) in terms of pasta and bread quality. Even if Monlis and Norberto are two diploid wheats lacking Glu-B1 genes, they had a high UPP% when compared with the other tetraploid cultivars. This suggests that UPP% is closely linked to Glu-A1, independently of Glu-B1. The high UPP% of Monlis and Norberto could be due to their late GFPs (even though they were short GFPs), resulting in more days with high temperatures (>30 °C) than the other cultivars. In fact, during the last stage of the GFP, as the grain dehydration progresses, the activity of glutathione reductase decreases and this leads to the formation of mixed disulfides between glutathione and glutenins, which is the UPP formation [28].

S2 was characterized by a longer GFP, greater water availability, and less days with maximum temperatures greater than 30 °C than S1. The low UPP% during S2 was likely a consequence of a shorter dehydration period. The decrease in UPP% was found independent to changes in total grain protein and gluten content [18].

Table 3 reveals that cultivar significantly impacted all grain protein fractions and ratios, while season and clipping had limited or unsignificant effects. Arzadun et al. [29] showed that even the quality of yield and forage after clipping were mainly influenced by cultivars. Clipping impacted soluble proteins, glutenins, and the gliadins/glutenins ratio (GLI/GLU) but did not have an effect on gliadins and the HMW/LMW ratio (HMW/LMW). Season only had significant effects on soluble proteins and glutenins. Remarkably, interactions were found to be highly significant for the majority of variables, underlining the fact that cultivars responded differently to clipping in each season.

Table 5 illustrates the variability in protein composition as a function of clipping and cultivar in both seasons. In the dry season of S1, clipping significantly reduced the gliadin content of Cappelli and Giovanni Paolo (by almost half), while it increased glutenin, albumin, and globulin contents, leading to a strong decrease in GLI/GLU (from 2.71 to 0.4 and 2.90 to 1.24, respectively). It has been reported that low GLI/GLU is associated with an increase in the dough resistance to extension, which could interfere negatively with the dough fermentation process (development time and stability) [19,30]. In fact, gliadins and glutenins differently influence dough rheological properties. In particular, monomeric gliadins impact dough viscosity and extensibility, while polymeric glutenins confer dough cohesivity and elasticity [19]. Few researchers have investigated the role of non-gluten proteins on the quality of flour, dough, and wheat-end products. In particular, globulins were found to improve the disulfide bond formation and protein aggregation under high temperatures (100 °C) [31].

Padre Pio had the highest HMW/LMW (above 0.30) in both the clipped and non-clipped treatments. High HMW/LMW was found to be related to low UPP%, weak dough and gluten quality, and thus poor bread making quality [27]. The HMW/LMW of Khorasan was impacted by clipping, without interfering with the total glutenin content. The protein fractions did not change as a function of clipping in the other cultivars, despite the stressful environmental conditions. This indicates their high ability to cope with these combined stresses (clipping and water deficit).

In the rainy season, S2, the protein compositions of Giovanni Paolo, Monlis, and Norberto were significantly influenced by clipping. Their albumins, globulins, and glutenins contents decreased by 40–60% and by 60–73%, respectively, in response to clipping. However, the gliadin contents of the clipped grains increased 1.9–2.9 times, leading to a 6–10 times increase in their GLI/GLU ratio. Under the non-clipped conditions, Giovanni Paolo, Monlis, and Norberto had a GLI/GLU ≤ 1, meaning that the tenacity of their dough exceeded the extensibility. This might negatively impact dough fermentation and thus limit bread volume. Therefore, the notable increase in GLI/GLU due to clipping makes them more suitable, at least for these dough traits, for fermented end-products compared to the non-clipped ones [19]. The protein fractions of Padre Pio did not differ significantly after clipping; however, the GLI/GLU decreased, and so its dough extensibility did too. Clipping also lowered its HMW/LMW to less than 0.30, improving its dough quality. The same applied to the HMW/LMW of Khorasan, while that of Giovanni Paolo increased to be over 0.30. A change in HMW/LMW means that the changes in the glutenin subunits, resulting in the overall glutenin change, were not of the same entity.

Overall, Giovanni Paolo was the only cultivar showing a significant effect of clipping to protein composition in both seasons. Probably, this might be correlated to its earliest and longest GFP (Table 2) when compared to the cultivars studied. In fact, the role of phenology is generally recognized as being one of the main drivers of adaptation and cultivar x environment interaction [29,32,33].

### 3.3. Relationship between Genotypic Variation in Grain Nitrogen Content and Protein Fractions

It has been shown that genotypic differences in the grain nitrogen content can account for at least a part of the variation in quality parameters and in the partitioning of total grain N between protein fractions [4]. To evaluate whether clipping affects these relationships, cultivar means were used to calculate the relationships between albumin and globulin, gliadins, and glutenins with EGNµg under clipped and non-clipped treatments. These relationships provided information about the strength of the relationship between genotypic variation in protein fractions and the EGNµg (coefficient of correlation and determination), and the extent of the variation in protein for each unit of variation in total grain N or partitioning coefficient (slope of the regression).

**Figure 3:**
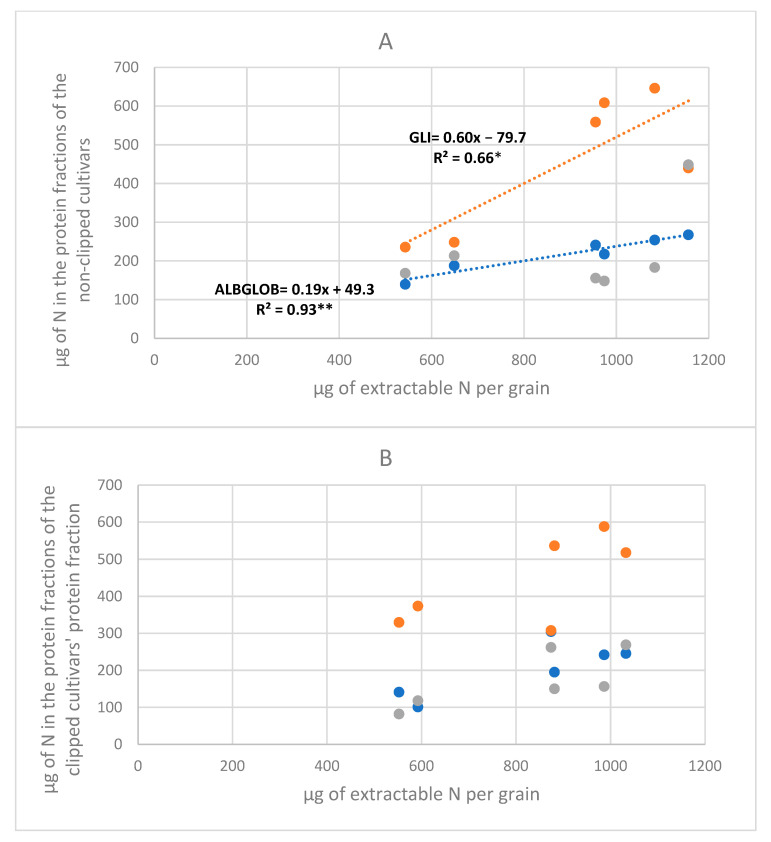
Relationships between the variation in the grain extractable nitrogen (x) and the N content of the protein fractions (y): albumin and globulins (ALBGLOB) (blue dots), glutenins (GLU) (gray dots), and gliadins (GLI) (orange dots), under non-clipped (**A**) and clipped (**B**) conditions. Points are cultivar means across seasons. ** *p* ≤ 0.01, * *p* ≤ 0.05. Non-significant relationships are not reported.

As shown in Figure 3, in both the non-clipped and clipped treatments, gliadins were present in larger amounts than glutenins, albumins, and globulins, in agreement with previous studies [4]. In the non-clipped treatment, the fraction of albumins and globulins was the one most tightly associated with EGNµg, which accounted for 93% of its genotypic variation, compared with 66% for gliadins. On the contrary, the genotypic polymeric glutenin content varied independently from EGNµg (R^2^ = 0.20 ns). Our results confirm previous results [4] on old durum wheats, where albumins and globulins were more associated with total grain N than the storage proteins, but contradict their results on glutenins also being associated with GNug. The high variability in the genes coding for glutenins between the cultivars selected could be behind the discrepancy in the results.

Gliadin was also the fraction that varied the most in response to the genotypic variation in EGNµg with a slope of regression almost three times higher than that of albumins and globulins (0.60 ± 0.39 vs. 0.19 ± 0.66 µg of N). This result is in accordance with what was previously found in a set of durum wheats [4]. This could be because albumins and globulins are sink limited, and therefore a variation in grain nitrogen is less likely to influence the quantity of N allocated to the albumins and globulins. On the other hand, even though the accumulation of gliadins and glutenins are limited sources, they could behave differently because most of the genes behind glutenin and gliadin regulation are diverse and perform differently [34]. This means that the variability existing between the cultivars affected the relationship between EGNµg and glutenins. Clipping interfered with this relationship because, under clipped conditions, the metabolic and storage proteins varied independently from total grain N. The lack of associations between protein formation and EGNµg under clipped conditions suggests that this technique could alter the partitioning of N to the different protein fractions. During grain filling, the albumins and globulins, as well as the gliadins and glutenins, do not start to accumulate together [21,35]. Consequently, any change in the GFP could differently affect their accumulation depending on the cultivar studied. Under non-clipped and clipped treatments, the variation in GLI/GLU was not associated with the variation in total grain N [8], confirming previous results [4] but contrasting the result by [36].

## 4. Conclusions

The primary objective of managing dual-purpose crops is to optimize the earnings generated by both harvested forages and grains, once ensuring that the grain quality remains unchanged. Moreover, the demand for ancient and old wheats and their derived products has been steadily increasing due to changing consumer preferences and the growing interest in traditional and heritage foods. Our results confirm that clipping may modify grain protein fractions, at least for the set of studied wheats and under the climatic conditions of this study. This could affect the grain’s quality traits and consequently the rheological properties of the dough and their suitability for food applications, mainly pasta and bread. Khorasan was the only cultivar not to be affected by clipping and to have an unchangeable protein composition (which could imply an unchanged quality of flour, dough, and end-product). For other cultivars, the effect of clipping on protein accumulation was significant and could be considered to be positive or negative depending on the climatic conditions of the season and on the type of the end-product desired. These results might be valuable for agronomists and farmers adopting or willing to adopt mixed farming practices under low-input conditions.

Capitalizing on the trend of cultivating ancient and old wheats and ensuring a high quality of forages, grain yield, and technological quality via the clipping technique can lead to expanded market reach and potential growth in sales volumes, thereby boosting overall farm revenues and profitability.

## Figures and Tables

**Table 1:** Characteristics of the studied wheats.

Species	Cultivars	Geographic or Genetic Origin	Year of Release
*Triticum durum* Desf.	Senatore Cappelli (Cappelli)	North-African landrace Jean Retifah	1920
*Triticum* *turanicum*	Khorasan	Near and Central Asian landrace Jakubz	2019
*Triticum monococcum* ssp *monococcum*	Monlis	Genealogical selection of local population	2006
*Triticum monococcum* ssp *monococcum*	Norberto	Genealogical selection of local population	Known before 2018 as ID331
*Triticum dicoccum* Schubler Improved Emmer	Giovanni Paolo	*Triticum dicoccum* Schubler line selected from Molise population × *T. turgidum* spp. *durum* Desf. cv Simeto	2008
*Triticum dicoccum* Schubler Improved Emmer	Padre Pio	*Triticum dicoccum* Schubler line selected from Molise population × *T. turgidum* spp. *durum* Desf. cv Simeto	2008

**Table 2:** Meteorological data during the grain filling of the six cultivars in the first (S1) and second (S2) season.

Cultivar	Onset of Grain Filling	Duration of Grain Filling (d)	Rainfall (mm)	Rainfall Deficit (mm)	Minimum Temperature (°C)	Maximum Temperature (°C)	N° of Days with 25 °C < T < 30 °C	N° of Days with T > 30 °C
**S1**								
Giovanni Paolo	15 March	57	16	−279	10	21	0	13
Cappelli, Khorasan, Padre Pio	15 April	41	1	−208	11	24	0	13
Monlis, Norberto	15 May	26	6	−164	14	26	2	20
**S2**								
Giovanni Paolo	1 April	59	240	−6	13	22	10	2
Cappelli, Khorasan, Padre Pio	20 April	49	238	17	15	24	15	4
Monlis, Norberto	20 May	36	73	−135	19	29	22	11

**Table 3:** Sum of squares percentage from ANOVA and significance of the F test for the effects of clipping, cultivar, season, and their interactions on extractable grain nitrogen (EGNµg), unextractable protein (UP%), and unextractable polymeric protein (UPP%) percentages, protein fractions, and ratios.

	Cultivar	Clipping	Season	Cultivar × Clipping × Season	Cultivar × Clipping	Clipping × Season
EGNµg	***	**	***	ns	ns	ns
	90.05	3.27	4.88	0.66	0.61	0.53
UPP%	***	ns	**	ns	ns	ns
	89.3	1.67	3.36	2.44	3.22	0
UP%	***	ns	***	ns	ns	ns
	84.92	0.54	9.45	2.86	1.6	0.63
µg of N in Albumins and Globulins	***	*	*	ns	***	**
	56.23	0.88	3.76	6.5	12.6	20.04
µg of N in Gliadins	***	ns	ns	***	***	***
	51.35	0.18	0.4	16.16	17.15	14.75
µg of N in Glutenins	***	**	**	***	***	***
	42.96	3.54	3.67	20.86	13.52	15.44
GLI/GLU	***	*	ns	***	***	***
	32.06	5.2	0.31	20.69	23.04	18.7
HMW/LMW	***	ns	ns	***	***	***
	74.63	0.36	0.1	6.62	15.25	3.04

ns: not significant; *: *p* ≤ 0.05; **: *p* ≤ 0.01; ***: *p* ≤ 0.001; SS: sum of squares. GLI/GLU: ratio of gliadins over glutenins; HMW/LMW: ratio of high-molecular-weight over low-molecular-weight glutenin subunits.

**Table 4:** Extractable grain nitrogen (EGNµg), unextractable protein (UP%), and unextractable polymeric protein (UPP%) percentages as a function of clipping, cultivar, and season. Values (mean ± standard deviation) in the same column flanked by different letters (a–e) are significantly different (*p* ≤ 0.05) based on Tukey’s test.

Factor	EGNµg	UP%	UPP%
Clipping	*	ns	ns
NC	893	8.12	29.3
C	814	8.41	29.8
Cultivars	***	***	***
Cappelli	929 b	6.37 c	33.9 bc
Giovanni Paolo	1095 a	10.6 a	36.6 a
Khorasan	1027 a	8.26 bc	31.4 d
Monlis	533 c	10.7 a	34.1 b
Norberto	614 c	9.32 ab	32.6 cd
Padre Pio	925 b	4.37 d	28.3 e
Season	***	***	**
S1	806	9.00	30.0
S2	902	7.54	29.2

ns: not significant; *: *p* ≤ 0.05; **: *p* ≤ 0.01; ***: *p* ≤ 0.001. NC: not clipped; C: clipped.

**Table 5:** Effects of clipping on the protein fractions and their ratios of the six cultivars in the first (S1) and second (S2) season.

	µg of N in Albumins and Globulins		µg of N in Gliadins		µg of N in Glutenins		GLI/GLU		HMW/LMW	
	NC	C	NC	C	NC	C	NC	C	NC	C
**S1**										
Cappelli	241	411	540	149	199	369	2.71	0.40	0.18	0.20
Giovanni Paolo	182	275	634	329	218	352	2.90	1.24	0.24	0.30
Khorasan	232	261	685	632	178	173	3.85	3.65	0.28	0.24
Monlis	76.0	79.0	248	247	100	97.0	2.48	2.53	0.25	0.24
Norberto	89.0	86.0	344	314	114	98.0	3.04	3.21	0.25	0.26
Padre Pio	206	196	600	515	115	87.0	5.20	5.96	0.32	0.34
LSD_0.05_	53.8		143		65.5		0.91		0.02	
**S2**										
Cappelli	241	198	577	466	112	155	3.75	4.18	0.20	0.19
Giovanni Paolo	353	216	246	707	679	186	0.37	3.82	0.30	0.35
Khorasan	276	223	607	544	188	140	3.23	3.88	0.27	0.21
Monlis	203	203	223	412	236	67.0	1.02	6.18	0.24	0.24
Norberto	286	116	152	433	313	138	0.48	3.15	0.25	0.26
Padre Pio	229	194	617	558	181	213	3.41	2.60	0.37	0.25
LSD_0.05_	41.8		64.2		63.7		0.39		0.09	

NC: non-clipped; C: clipped; GLI/GLU: ratio of gliadins over glutenins; HMW/LMW: ratio of high-molecular-weight over low-molecular-weight glutenin subunits. LSD_0.05_: least significant difference (*p* ≤ 0.05) for the comparison of means.

## Data Availability

The data used to support the findings of this study can be made available by the corresponding author upon request.

## Supplementary Materials

The following supporting information can be downloaded at https://www.mdpi.com/article/10.3390/foods12132582/s1: Figure S1: RP-HPLC chromatograms representing the area under the curve of the gliadins during the 60 min of separation of the six cultivars studied, Padre Pio, Norberto, Monlis, Giovanni Paolo, Kamut, and Cappelli. Figure S2: RP-HPLC chromatograms representing the area under the curve of the glutenins during the 60 min of separation of the six cultivars studied, Padre Pio, Norberto, Monlis, Giovanni Paolo, Kamut, and Cappelli.
