# Supplementary material for: Clipping Effect on the Grain Nitrogen and Protein Fractions of Ancient and Old Wheats Grown in a Mediterranean Environment
Source: Foods. 2023 Jul 2;12(13):2582. doi: 10.3390/foods12132582 (PMC10341193; doi:10.3390/foods12132582)
Supplement: Supplementary file 1 [file foods-12-02582-s001.zip › foods-2454589-supplementary.pdf]

foods

MDPI

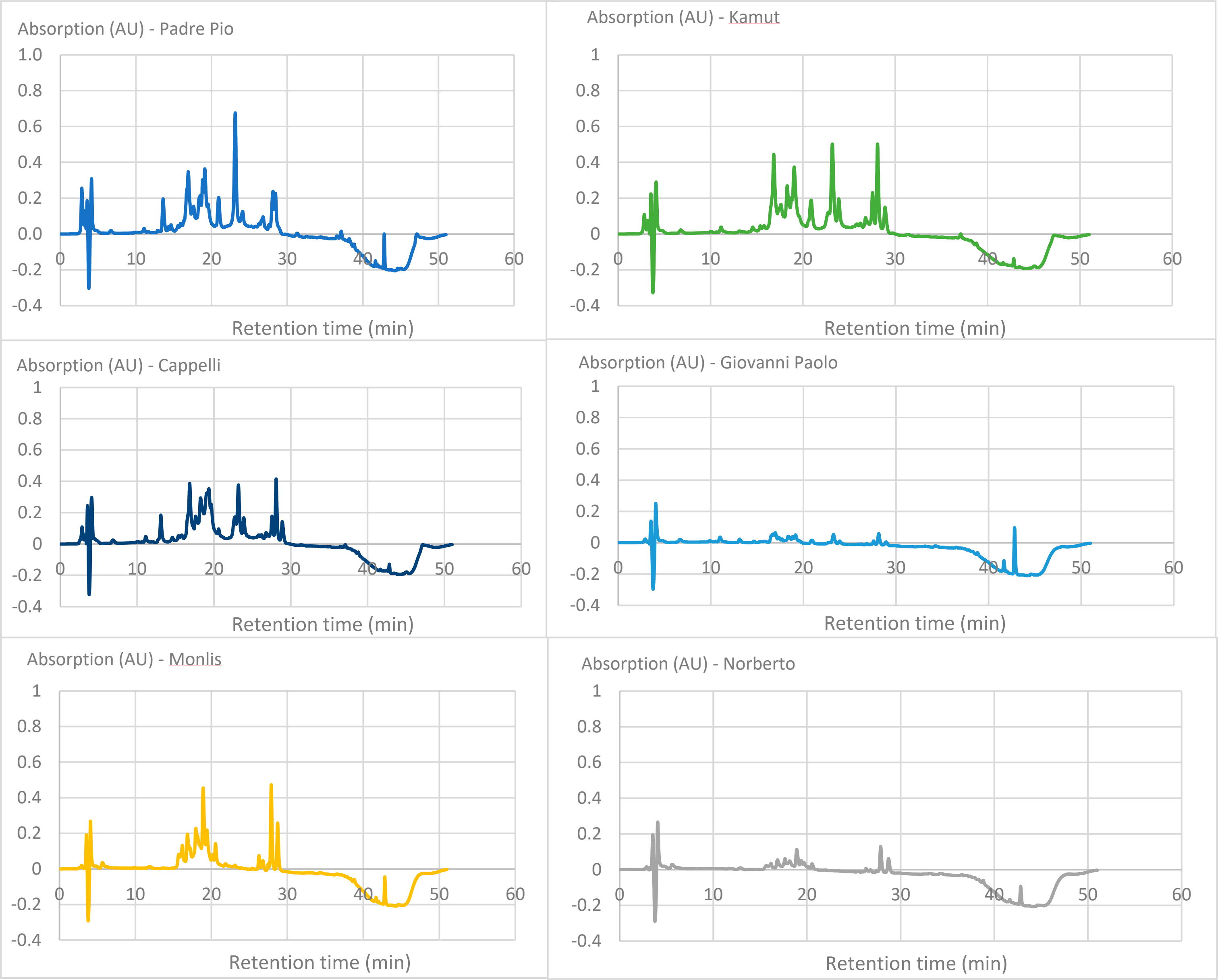

Figure S1. RP-HPLC chromatograms representing the area under the curve of the gliadins during the 60 minutes of separation of the six cultivars studied, Padre Pio, Norberto, Monlis, Giovanni Paolo, Kamut and Cappelli.

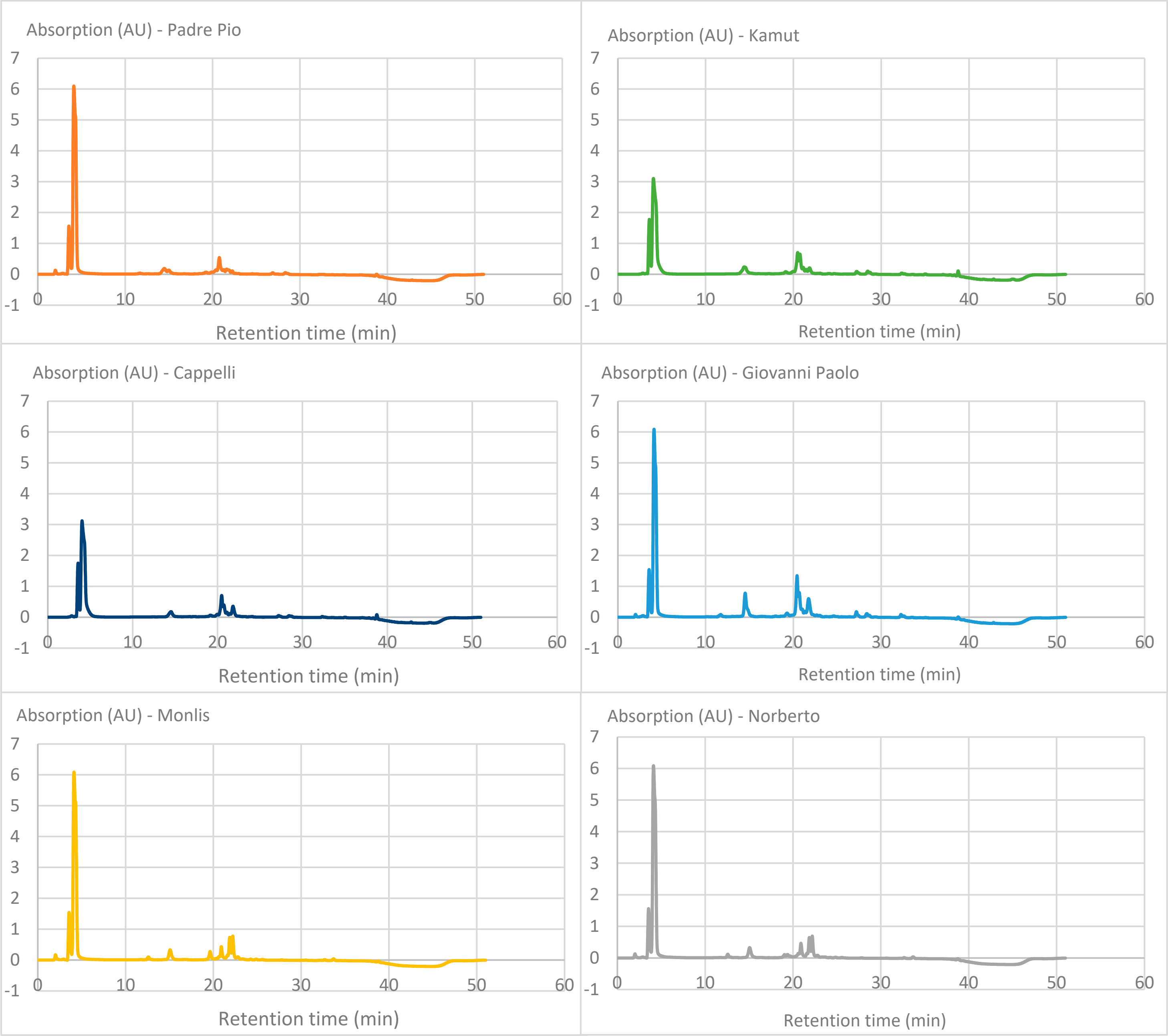

Figure S2. RP-HPLC chromatograms representing the area under the curve of the glutenins during the 60 minutes of separation of the six cultivars studied, Padre Pio, Norberto, Monlis, Giovanni Paolo, Kamut and Cappelli.
